# The Influence of Human Body Orientation on Distance Judgments

**DOI:** 10.3389/fpsyg.2016.00217

**Published:** 2016-03-09

**Authors:** Edgard Jung, Kohske Takahashi, Katsumi Watanabe, Stephan de la Rosa, Martin V. Butz, Heinrich H. Bülthoff, Tobias Meilinger

**Affiliations:** ^1^Max Planck Institute for Biological CyberneticsTübingen, Germany; ^2^University of InnsbruckInnsbruck, Austria; ^3^Research Center for Advanced Science and Technology, The University of TokyoTokyo, Japan; ^4^Waseda UniversityTokyo, Japan; ^5^Eberhard Karls UniversityTübingen, Germany; ^6^Department of Brain and Cognitive Engineering, Korea UniversitySeoul, South Korea

**Keywords:** distance perception, body orientation, proxemics, virtual reality, avatar

## Abstract

People maintain larger distances to other peoples’ front than to their back. We investigated if humans also judge another person as closer when viewing their front than their back. Participants watched animated virtual characters (avatars) and moved a virtual plane toward their location after the avatar was removed. In Experiment 1, participants judged avatars, which were facing them as closer and made quicker estimates than to avatars looking away. In Experiment 2, avatars were rotated in 30 degree steps around the vertical axis. Observers judged avatars roughly facing them (i.e., looking max. 60 degrees away) as closer than avatars roughly looking away. No particular effect was observed for avatars directly facing and also gazing at the observer. We conclude that body orientation was sufficient to generate the asymmetry. Sensitivity of the orientation effect to gaze and to interpersonal distance would have suggested involvement of social processing, but this was not observed. We discuss social and lower-level processing as potential reasons for the effect.

## Introduction

Distance is a social and spatial property. One feels “close” to somebody; another person can be distant in a spatial and a social sense. These metaphors parallel findings in work on interpersonal distance which is the distance humans keep to each other. For example, people maintain larger distances to people they dislike (Kleck, 1968) or that were described as immoral (Iachini et al., 2015), but shorter distances to people they feel connected to (Willis, 1966; Patterson, 1977). People usually maintain a space of approximately 1.2 m around themselves (Hall, 1968), which, if violated, can lead to anxiety, physiological arousal and defensive acts (Felipe and Sommer, 1966). Studies by Hayduk (1981) have shown that this personal space is not a circle; people maintain larger distances to the front than to the back of other people. These results were replicated in virtual environments with virtual characters (Bailenson et al., 2003).

If people keep larger distances to the front of other people than to their back, the question arises: do interpersonal distance differences only exist in behavior or is the distance to humans also perceived differently: do people perceive other persons as closer when they face their front than their back? Answering this question is the main motivation of the present work.

The second motivation targets the underlying processes. The body orientation effect on distance perception –if existing – may originate from social processes or from lower-level object-orientation-dependent processes. Some indications come from a study that showed that distance perception can be influenced by the orientation of a neutral object (Takahashi et al., 2013). Participants saw cones floating around at certain distances, either facing an observer or facing away. Facing objects were estimated as closer. Results indicate, firstly, that an effect of object orientation on distance perception exists, and secondly, that this effect works also with non-human objects. However, as the cones were self-propelled and changed their trajectory as well as orientation (always facing the observer), participants might have considered these objects as animate and estimations might have depended on processes ascribed to intentional beings, i.e., social processes.

Social influences on perception seem possible. Several studies described social, cognitive, or affective influences on perception (for a summary see Proffitt and Linkenauger, 2013; Balcetis, 2015; for critical evaluation see Firestone and Scholl, 2015a). For example, when people are facing a hill, those in a happy mood perceive the hill as less steep compared to those in a sad mood (Riener et al., 2011). Similarly, when in fear the hill looks more steep (Stefanucci et al., 2008). In addition, the perception of steepness is influenced by the age of observers (Bhalla and Proffitt, 1999), their level of fatigue (Proffitt et al., 1995), the weight of their rucksack (Proffitt et al., 2003) and the quality of their relationship with the person who accompanies them (Schnall et al., 2008). Desired objects are seen as closer than undesirable objects (Balcetis and Dunning, 2009), and people estimate desired locations as closer (Alter and Balcetis, 2011). Also threatening objects (such as a tarantula) are perceived as closer than neutral or non-threatening objects (Cole et al., 2013). Perceiving a threatening object as closer may provide an individual with more time to react to the threat, thus be an adaptive mechanism that enhances survival (Haselton et al., 2005). Similarly, estimating the steepness of a hill as more steep when scared or in a fatigued state may act as a protective mechanism, to avoid risks and possible injuries (Proffitt, 2006a).

If social processes indeed underlie body orientation differences in perception we predicted two effects: a modulation of perceptual asymmetry with distance and an effect of gaze. In case the body orientation effect relates to cognitive processes underlying interpersonal distance regulation (i.e., social processes) the effect might be more pronounced at interpersonal distance than at distances clearly shorter or larger. Takahashi et al. (2013) indeed observed a sensitive distance interval in which the effect was observed. Finding the same results with human bodies would suggest a relation to processes involved in interpersonal distance and therefore social processes.

A second indicator for social processing is gaze. Joint attention, i.e., estimating the gaze of another person and aligning one’s own gaze to that seems to be an early developing basis for multiple social capabilities (Thomasello, 1995). Being gazed at or not was also shown to influence interpersonal distance (Bailenson et al., 2001, 2003). As a consequence we will examine whether an effect of body orientation on distance perception depends on being gazed at which would indicate social processing.

The main motivation of the present work was to examine whether effects of body orientation on interpersonal distance are already found in the perception of a human body. Furthermore, we examined if such an effect was modulated by distance and depended on gaze, both of which would suggest that the effect is social in nature.

## Experiment 1

In Experiment 1, we presented avatars that were either facing an observer or were facing away, in order to measure the orientation effect in the distance perception of a human body. We predicted the facing conditions to be estimated as closer.

### Method

#### Participants

Fifteen volunteers (five females) took part in the experiment and were paid 8 Euro/hour for participating. The mean age was 27.8 years (*SD* = 7.45). All participants gave informed written consent before conducting the experiment. The study was approved by the ethics committee of the University Clinic Tübingen.

#### Setup

**FIGURE 1:**
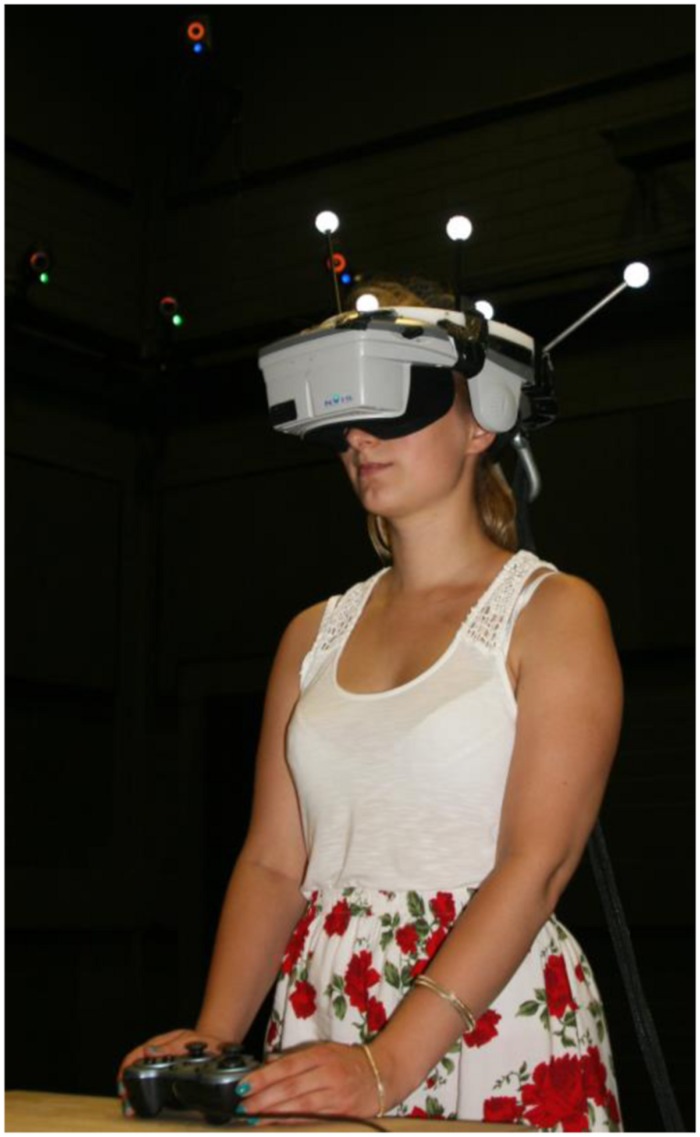
**A participant equipped with a head mounted display whose orienation was tracked by motion tracking cameras.** Participants used a joystick fixated to a table to adjust their distance estimates.

As shown in **Figure 1**, participants stood in front of a table on which a standard Gamepad (Logitech F510) was mounted and thus kept a constant body location and orientation during the experiment. They experienced the virtual environment through an nVisor SX60 HMD. with a resolution of 1280 pixel × 1024 pixel for each eye and a field of view of 44° (horizontal) × 35° (vertical) with 100% overlap. We fixed interpupillary distance at 6.5 cm. Participants’ head position was tracked by 20 high-speed motion capture cameras with 120 Hz (Vicon^®^MX 13 and Vicon^®^T160) to render an egocentric view of the virtual environment in the HMD in real-time on a NVIDIA GeForce GTX 980. The experiment was programed in Unity 4.3.

#### Stimuli

**FIGURE 2:**
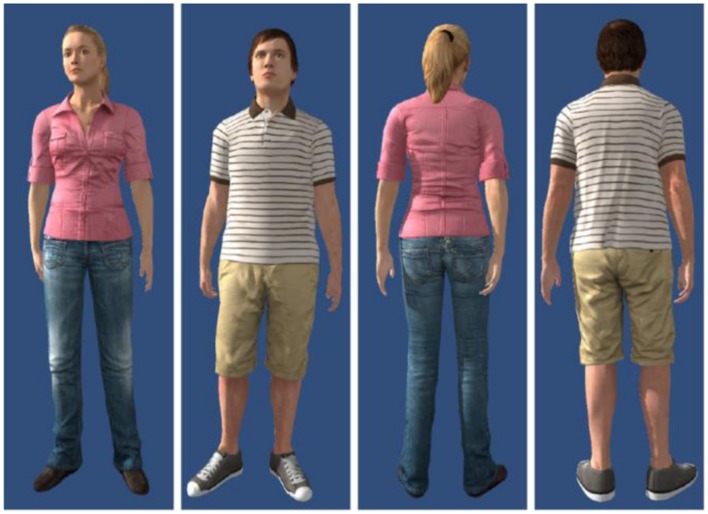
**Front and back view of the male and female avatars.** The blue background is equal to the free space the avatars were presented in.

Two avatars, one male and the other female, were used (**Figure 2**). The male avatar had a height of 175 cm and the female avatar had a height of 165 cm. During presentation the avatars were animated with the same animation swaying left and right slightly to enhance realism. The midpoint of the avatars was determined in a pre-experiment, where 10 participants watched the animated avatars from the side and moved a plane to the center of the avatar. The starting point of the plane was to the left and right of the avatar and the procedure was repeated for the avatars facing to the left or right. The midpoint of each of the two avatars was determined by averaging across all situations. This midpoint point worked as the location of the avatar and the pivotal axis around which it was rotated facing the participants or looking away. When facing a participant, the avatars constantly looked at the participant’s head and resulted in changes in eye direction during avatar movement and/or participant movement to the side. We presented the avatars at five different distances (0.5, 1, 2, 4, and 8 m) and three different heights (at ground level, 10 cm higher, 10 cm lower). Varying height prevented participants from using simply the visual angle to the feet of the avatar that would be an informative distance cue when all avatars stood on the ground place (Proffitt, 2006b).

During the distance estimation, a 10 m × 10 m plane with a green camouflage pattern appeared. Participants used the sliding stick of the gamepad to move the plane forward or backward. In even number trials the plane appeared 30 cm in front of the participants, in odd number trials 12 m in front of the participants. Except for the avatars and the plane no visual cues were presented. We used a blue background (**Figure 2**) and a directional light source came from the left in addition to ambient lighting.

#### Procedure

First, participants received detailed written and oral description of the task. They trained the task on example trials as long as they wished before proceeding to the real experiment. Every trial began with the presentation of the avatar for 5 s. The avatar disappeared and the plane was shown. Participants moved the plane to the avatar’s former location and pressed a button at the gamepad to confirm their choice. Participants were instructed to act as accurately and quickly as possible. The trial ended and a black screen with “continue with a click” followed. This could be used for a rest and participants could take a break anytime after completing a trial. We recorded the estimated distance and latency (i.e., time between plane appearance and button press). Furthermore we recorded head position throughout the experiment.

#### Design

We used a 2 (orientation) × 5 (distance) × 3 (height) × 2 (avatar sex) fully balanced within design. This resulted in 60 trials that we repeated three times, resulting in 180 trials altogether. Each block of 60 trials was presented in a newly determined random order. As we were not interested in the avatar sex as such but wanted to guarantee a minimum of diversity of avatars, avatar sex was not analyzed as an experimental factor. However, including avatar sex into the analysis did not change the pattern of the reported results.

### Results

**FIGURE 3:**
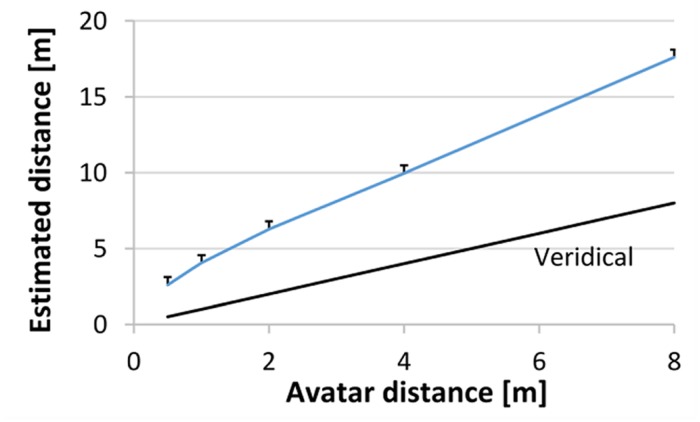
**Estimated distance of the participants as a function of distance.** Means and standard errors as estimated from the marginal means are shown.

The data was analyzed with a linear mixed model with the factors orientation (two levels), height (three levels) and distance (five levels). We calculated the estimation error by subtracting the estimated distance from the actual distance. Errors or latencies deviating more than 3*SD* from the overall mean were not analyzed (<2.5%). In general, participants had an error larger than 0, so they overestimated the distances as indicated in the significant intercept*, F*(1,14) = 99.34, *p* < 0.001. As shown in **Figure 3** the overestimation became larger with larger distances, *F*(4,2607) = 869.24, *p* < 0.001, $\eta_{p}^{2}$ = 0.97, and the participants estimated larger distances distance more quickly, *F*(4,2530) = 6.54, *p* < 0.001, $\eta_{p}^{2}$ = 0.13.^1^

**FIGURE 4:**
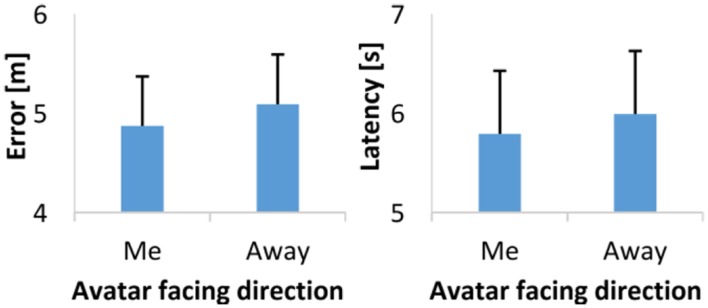
**Facing avatars led to closer estimations and quicker reactions**.

Our main interest was the effect of facing orientation on distance perception. Indeed, we found a main effect of orientation, *F*(1,2607) = 6.30, *p* = 0.012, $\eta_{p}^{2}$ = 0.23. The participants estimated the avatars facing them on average as 22 cm closer than the avatars facing away (**Figure 4**). Also, the participants reacted more quickly when faced by the avatar than when the avatar was looking away, *F*(1,2530) = 4.39, *p* = 0.036, $\eta_{p}^{2}$ = 0.51. The orientation effect in error was modulated by height, *F*(2,2607) = 4.36, *p* = 0.013, $\eta_{p}^{2}$ = 0.30. The difference between facing orientations was only significant for the avatars at the ground level *F*(1,863) = 9.65, *p* = 0.002, and for the elevated avatars, *F*(1,855) = 4,66, *p* = 0.031.

The second motivation was to examine if the orientation effect was based on social processing. A sensitive distance for the orientation effect around the interpersonal distance (or its projection within the virtual space) would point toward social processing. However, the orientation effect was not modulated by distance neither for error, *F*(1,2607) = 2.01, *p* = 0.090, $\eta_{p}^{2}$ = 0.08, nor for latency, *F*(1,2530) = 1.61, *p* = 0.168, $\eta_{p}^{2}$ = 0.09. No support for social processing could be found.

We also found a main effect of height in error, *F*(2,2607) = 30.31, *p* < 0.001, $\eta_{p}^{2}$ = 0.69, and latency, *F*(2,2530) = 4.85, *p* = 0.008, $\eta_{p}^{2}$ = 0.25. The higher the avatars were presented, the larger participants estimated the distance. Fastest button presses were found at the ground level. There was a three way interaction between orientation, height and distance for error, *F*(8,2607) = 2.73, *p* = 0.005, $\eta_{p}^{2}$ = 0.16, and latency, *F*(8,2530) = 2.73, *p* = 0.005, $\eta_{p}^{2}$ = 0.21, which, however, did not invert the main effect of orientation. No other effects attained significance.

### Discussion

Behaviorally, people maintain larger distances to other people who are facing toward them (Hayduk, 1981; Bailenson et al., 2003). Our results showed that the participants perceived the avatars facing them as closer and reacted quicker to them than to the avatars looking away. This difference in distance judgment may precede interpersonal distance regulation. People adjust their interpersonal distance to others to the comfortable range (Hayduk, 1981). When perceiving others’ fronts as closer than their backs, then people have to keep larger physical (or virtual) distances to others’ fronts than backs. The orientation effect in perception thus would lead to the orientation effect in distance regulation.

The participants reacted faster when faced by the avatar. This boost could be explained by enhanced attention. Several studies showed an increase in attention and reaction time to the front of a body as compared to its back. (Bosbach et al., 2004; Shi et al., 2010), even for 6 months old infants (Bardi et al., 2015). Our reaction time differences are well consistent with the literature.

We also observed the effect of height. Even though we used different elevation levels and no ground plane to minimize the reliability the visual angle toward avatars’ feet as a distance cue (Proffitt, 2006b), the participants seemed to have relied on it. The interaction between height and orientation indicates that the effect of orientation was strongest and in fact only significant for the avatars presented at the ground level or higher. Maybe small or lower interaction partners are on average less dangerous and thus do not inflict a distance estimation bias, which could prevent potential future harm. Or lower avatars do not inflict the impression of approaching so much and are therefore not estimated as closer. However, without further evidence, we can only speculate of why this was the case.

In case the body orientation effect was related to processing of interpersonal distance and thus social processing there should have been a sensitive distance around the typical interpersonal distance or its projection within virtual space. However, the body orientation effect did not interact with distance. No support for social processing was found in this way.

The participants clearly overestimated the distances. Typically, people underestimate distances in virtual environments (for a detailed review see Loomis and Knapp, 2003) and the observed overestimation in the present data seems initially surprising. However, we do think that the general distance compression in virtual environments might explain our observations. The avatars provided several distance cues. For example, they had a familiar size, they moved naturally and thus probably led to less underestimation (Mohler et al., 2010). Contrarily, the plane used for estimation provided fewer distance cues. It had no depth structure, or familiar size cues. Fewer usable distance cues presumably led to more underestimation than with the avatars. Participants’ task was to match the perceived distance of the plane to the perceived distance of the avatar. With a larger underestimation of plane distance than avatar distance, subjective equal distances of the two can only be obtained when the plane is further away than the avatar (in the virtual space).

Distance estimation was faster for larger distances. This result is probably an artifact of the setup. Participants adjusted their decision a lot in closer conditions. They moved the plane forward and backward until they were satisfied and the plane matched with their judgment. This adaption took some time. At longer distances they did not fine-tune their estimates as much. This difference fits with the Weber–Fechner law (Hecht, 1924) that the sensitivity for differences is proportional to the stimulus size. At closer distances participants are more sensitive to small differences and invest more time in fine tuning the plane than at larger distances.

## Experiment 2

We observed an orientation effect of avatars both in the reaction time and in the estimated distance. In case this effect is social in nature we predicted that it depended on a highly social cue such as gaze. Alternatively, gaze might have been irrelevant and only the overall orientation of the body mattered. In order to investigate this question, we examined distance perception to avatars in multiple other facing orientations, not just front and back. Gaze could only be determined at frontal facing orientation, but not for rotated avatars which, however, still faced the observer with their body. If the gaze direction was crucial, an orientation effect should only be observed between frontal facing orientation (including gaze) and other orientations. If the body orientation was sufficient, all body orientations roughly facing an observer should be perceived as closer comparted with body orientations facing away.

### Methods

#### Participants

Fifteen volunteers (five females) were newly recruited and took part in the experiment and were paid 8 Euro/hour for participating. The mean age was 28.5 (*SD* = 7.16).

#### Materials and Procedure

**FIGURE 5:**
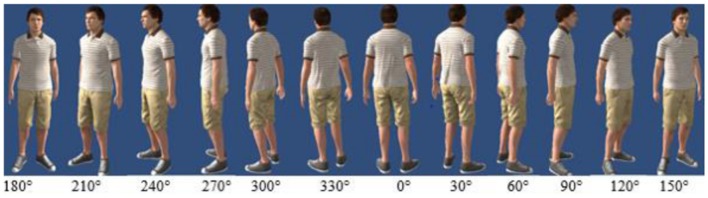
**Body orientations tested in Experiment 2**.

In Experiment 2, we kept all methods identical to those in Experiment 1 except the followings. The avatars were rotated in 30-degree steps around themselves in both directions (**Figure 5**), which resulted in 12 different body orientations (0, 30, 60, 90, 120, 150, 180, 210, 240, 270, 300, and 330°). In the 180° condition, the avatar faced the participants directly and in the 0° condition, the avatar had the same body orientation as the observer and therefore only the back was visible for the participants. As we were not interested in left vs. right body turns we pooled across left and right turns of equal size (i.e., 330 and 30°, 300 and 60°, 270 and 90°, 240 and 120°, 210 and 150°). This procedure resulted in seven orientation conditions (0, 30, 60, 90, 120, 150 and 180°). In order to equate the number of trials in all conditions 0 and 180° body orientations were presented twice as often as, for example, 330° which was pooled with 30°. This manipulation resulted in a 7 (orientation) × 5 (distance: 0,5, 1, 2, 4, and 8m) × 2 (avatar sex) × 3 (height) factorial design. In case an avatar fixating the observer was the crucial factor we expected shorter distance perception only at 180°, but not at other orientations. Alternatively, if body orientation was crucial we expected a difference between body orientation roughly facing a participant (i.e., 120, 150, 180, 210, and 240°) as compared with body orientations facing away (i.e., 300, 330, 0, 30, and 60°).

### Results and Discussion

**FIGURE 6:**
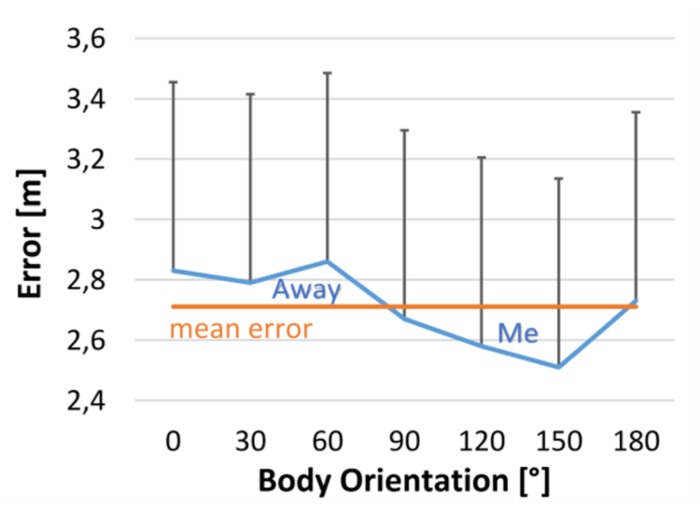
**Distance estimation error as a function of body orientation.** Left and right turns were pooled.

As in Experiment 1 we found general overestimation *F*(1,14) = 19.36, *p* = 0.001 and an effect of distance in error, *F*(4,2940) = 296.52, *p* < 0.001, $\eta_{p}^{2}$ = 0.60, and latency, *F*(4,2938) = 4.14, *p* = 0.002, $\eta_{p}^{2}$ = 0.09. The participants were quicker at larger distances and showed larger overestimations. **Figure 6** shows the effect of avatar orientation on the estimated distance. Although orientation did not lead to a significant main effect of body orientation, *F*(6,2940) = 1.35, *p* = 0.231, $\eta_{p}^{2}$ = 0.10, the pattern of results clearly excluded the possibility that only avatars gazing at an observer (i.e., the 180° condition) showed an effect. This would have resulted in the reverse pattern observed with smaller error (less overestimation) at 180° as compared to all other body orientations. Alternatively, rough body orientation might be the more important cue. Indeed, the pooled front conditions were estimated as closer than the back conditions, *F*(1,2578) = 5.63, *p* = 0.018, $\eta_{p}^{2}$ = 0.34. This result is consistent with the results of Experiment 1 and suggests that body front was the relevant cue to the orientation effect. No other effects reached significance. The effect of front-back body orientation was also not modulated by distance neither for error, *F* < 1, nor for latency, *F* < 1.

The results of Experiment 2 replicated the effect of orientation found in Experiment 1. Also the magnitude of perceived average difference in distance between facing and looking away conditions was similar (i.e., about 22 cm). The orienation effect depended on the virtual body’s orientation, but not directly on being gazed at. This suggests that the orientation effect is not based on gaze alone, but that body orienation is the more relevant contributor. In case the body orientation effect was social in nature, this would have suggested that gaze was relevant, which was not the case. Similarly, social processing would also go along a sensitive distance for the effect. However, also no interaction with distance was observed. In summary, we observed no support for social processes underlying the body orienation effect.

Please note that body orienation included also the face. We cannot tell whether the face, the torso, or both were responsible for the observed differences. Future experimentation has to differentiate between them.

Can the orientation effect be explained by the participants orienting themselves on the body part closest to them (e.g., the nose vs. the back)? We do not think this is a valid explanation. The observed difference of 22 cm is much larger than any potential difference of a nose sticking out relative to a back in frontal vs. backward facing conditions. Furthermore, in Experiment 2 avatars from the 90° conditions standing oblique to the participants were closer to the participants with their closest body part (arm/foot) than the nose or back in the 0 and 180° conditions of facing to vs. away from the participant. However, despite being closer with the closest body part these conditions were not judged as closer. Consequently, we think the explanation has to rely on other aspects.

## Experiment 3

In Experiment 2 distance estimation varied as a function of body orientation rather than gaze. However, even avatars fixated participants only in the 180° conditions, participants might have felt gazed at also in similar body orienations. In that case gaze rather than body orientation might still be the crucial factor. In order to examine this possibility we asked 10 naïve participants to rate the amount they felt gazed at with with the poles 0 “not at all” and 7 “maixmally gazed at.” Participants saw female and male avatars located at 2 m distance at ground level in all 12 body orientations presented exactly as in Experiment 2. They gave verbal estimates of their impression.

**FIGURE 7:**
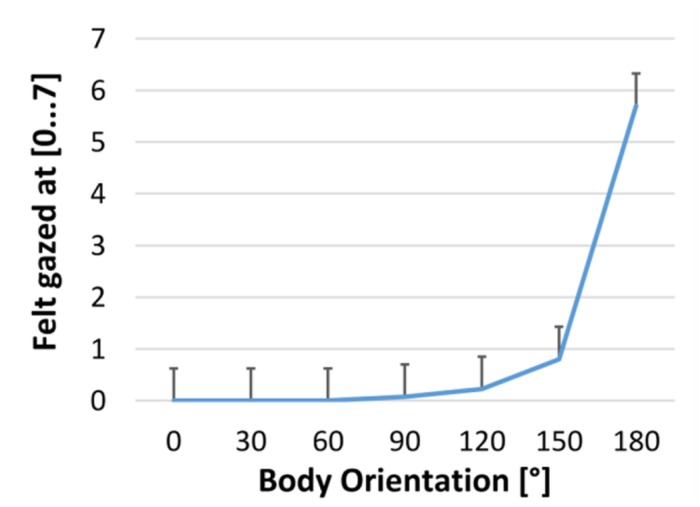
**Impression of being gazed at for avatars in different body orienations from not at all (0) to maximally (7)**.

**Figure 7** shows their average ratings which differed between body orienations, *F*(6,244) = 235.3, *p* < 0.001, $\eta_{p}^{2}$ = 0.93. The impression of being gazed at was clearly stronger for avatars facing a participants (180°) than at any other body orientation, *F*’s > 294, *p*’s < 0.001. At a much lower level, which only marginally differed from 0 “not gazed at,” *F*’s < 3.6, *p*’s > 0.090, the impression of being gazed at was still stronger for 150° than 120° body orientation, *F*(1,69) = 16.7, *p* < 0.001, which itself was stronger than 90°, *F*(1,69) = 4.93, *p* = 0.030.

The impression of being gazed at for avatars directly facing a participant (180°) was five points stronger than at any other body orientation. This difference comprises 70% of the whole available scale. This result clearly supports our conclusion from Experiment 2 that being gazed at is not the relevant cue underlying the effect of body orienation on distance perception. In this case distance estimation should have been shortest and at 180° and increased considerably from that. This was not the case.

Please note that we only exclude being gazed at as a crucial factor. Being within the visual field of another person – which applied in our case for body orienations up to 90 and 270°- might still be relevant to the effect. Nevertheless, this should only apply for distance perception to avatars and not not to similar results with objects (Takahashi et al., 2013) as objects do not have eyes or a visual field themselves.

## Experiment 4

Experiments 1 and 2 relied on a plane for distance estimation. However, prior work using objects employed a virtual sphere for estimating distances (Takahashi et al., 2013). Experiment 4 was conducted as a control for the estimation process. Participants extimated the distance with a sphere of 25 cm diameter floating at 1 m height above the invisible ground plane. Results from Experiments 2 and 3 suggested that body orientation rather than gaze was important for the effect of body orientation. Therefore, we only varied body orienation between front and back as in Experiment 1 and had the avatars always look straight ahead. When facing a participant the avatar looked clearly toward the participants, but did not follow their head movements. All other methods were identical with Experiment 1.

Twenty-one volunteers participated (10 female; mean age 26 years *SD* = 6 years). Contrary to Experiments 1 and 2, avatar orientation did not influence participants’ distance judgements *F*(1,3666) = 1.08, *p* = 0.299, $\eta_{p}^{2}$ = 0.08, nor their reaction time, *F* < 1. Consistent with Experiments 1 and 2 we observed an effect of distance in error *F*(4,3666) = 201, *p* < 0.001, $\eta_{p}^{2}$ = 0.33, and latency *F*(4,3669) = 101, *p* < 0.001, $\eta_{p}^{2}$ = 0.56. Again participants showed larger distance errors and quicker responses for larger distances. However, we did not observe a general distance overestimation, *F* < 1. We also found an effect of height in error *F*(2,3666) = 4.77, *p* = 0.009, $\eta_{p}^{2}$ = 0.20. However, contrary to Experiment 1 participants estimated higher avatars as closer and not as further away. Finally, there was an interaction between height and distance in latency *F*(8,3669) = 2.49, *p* = 0.011, $\eta_{p}^{2}$ = 0.10, suggesting slower reaction to low avatars close by and quicker reaction for ground level avatar at far distances.

Following subjective reports we speculate that many participants used a strategy of fixating the presumable floating height of the sphere at an avatar with the eyes and then keeping the gaze constant while moving the sphere forward or backward until it intersected with the gaze. This would explain why we did not find an orientation effect and no general overestimation as in Experiments 1 and 2. Overcompensating for the clearly visible avatar heights would explain why the height effect reversed. Participants knew they would have to fixate at a lower body location for avatars above the ground plane and overdone that correction resulting in a lower fixation and shorter estimated distances and the reverse pattern in avatars presented lower. In any case results indicate that the orientation effect in Experiments 1 and 2 depended on the estimation process. Using a plane for distance estimation seemed to have allowed also subtle influences to play a role. With clear visual gaze strategy these effects might have been overriden. Please note that a gaze strategy was not possible in the experiments from Takahashi et al. (2013) in which the sphere was moved toward the center of a virtual cube within which objects moved. There was not fixation point available.

## General Discussion

The orientation of an object or human body was shown to influence the interpersonal behavior of distance regulation (Hayduk, 1981; Bailenson et al., 2003), as well as distance perception to objects (Takahashi et al., 2013). Results from Experiments 1 and 2 extend this work into showing that there is also an orientation effect in distance judgments to a human body: human characters facing an observer were judged as closer than characters facing away.

Is the orientation effect on distance judgments a social effect or is it more due to lower-level object properties? We cannot fully answer this questions based on the present results. However, if social processing was involved we would have expected a modulation of the effect with distance and an effect of eye gaze neither of which we observed. Interpersonal distance of roughly 1.2 m (Hall, 1968) is influenced by various social cues such as how much a participant likes the other person (Kleck, 1968) or whether this person was described as moral or immoral (Iachini et al., 2015). If the observed body orientation effect was mainly present around this distance or the projection of this distance within the perceived virtual space, then this would have been an indication that the underlying effect was related to social processes. However, we did not observe an interaction between body orientation and testing distance in neither experiment. Consequently, there was no support for social processing from this side.

Showing the relevance of gaze for the orientation effect would have also indicated social processing. Estimating the gaze of others is an early developing core social capability (Thomasello, 1995) and being gazed at or not was also shown to influence interpersonal distance (Bailenson et al., 2001, 2003). However, even though participants felt gazed at mainly when the avatars’ eyes were directly fixating them in the 180° condition of frontal body orientation as indicated in Experiment 3, the asymmetry in distance judgments in Experiment 2 was not between this and other body orientations, but roughly between the body’s front vs. back. Thus, body orientation and not gaze seemed to be the crucial factor.

Relevance for gaze and effect modulation with distance would have been an indicator for social processing. Both predictions were not supported, however. Please note that the lack of evidence for social processing is not necessarily evidence against social processing, though. It is very well possible, that social processing still generate the effect. For example, humans just as almost all animals have a front and a back and one interacts with their front rather than their back side. It might be possible that potential interaction or looming yielded front sides as perceptually closer. Such a potentially “social” effect may be limited to animate entities, which move out of their own and are not (only) pushed by surrounding physical influences. Consequently, another prediction for social processing is dependence on animacy. The present experiments used moving human avatars. Prior studies relied on virtual cones, which moved through space abruptly changing their direction of movement (Takahashi et al., 2013). Such movement trajectories indicate self-propelled motion, which are typical for beings with own intentions (Schultz and Bülthoff, 2013) and thus beings that are capable of social interactions. It is subject to future research to examine if the perceived animacy of human characters or objects is a precondition for the orientation effect.

In case the orientation effect was not based on social processing, but rather on lower-level object processing, what could be the relevant processes? Maybe attention guidance could explain the effect. Attention is focused on the front rather than the back of a body (Bosbach et al., 2004; Shi et al., 2010; Bardi et al., 2015) or arrows (Hommel et al., 2001; Kuhn and Benson, 2007). When faced by an avatar or object, participants attention may thus be focused on locations before the avatar and therefore closer to the participant than when the avatar is facing away. Closer attentional focus may then have yielded shorter distance estimates. Future experimentation will have to examine the involvement of attention.

Social vs. lower-level processing is related to the question of whether the effect of body orienation on distance judgments is cognitive or perceptual in nature. The growing body of studies on social, emotional, physiological, and cognitive influence on perception (e.g., Proffitt and Linkenauger, 2013; Balcetis, 2015) have been criticized for not relating to perceptual processes at all (Firestone and Scholl, 2015a). Cognitive (Firestone and Scholl, 2014) and social (Durgin et al., 2009) influences on judgments as well as memory processes (Firestone and Scholl, 2015b) were used as alternative explanations to perceptual processing. We do not argue whether the orientation effect on distance is perceptual in nature or not as we think this is not possible based on the present data. Using “distance judgment” for our dependent variable is in that sense a neutral term which reflects that participants made a judgment, but should not be interpreted being either cognitive or perceptual. It is for future experimentation to decide this.

Body orientation was shown to influence interpersonal distance (Hayduk, 1981; Bailenson et al., 2003), while object orienation was shown to influence distance judgments (Takahashi et al., 2013). The present experimentation generalize the object orientation effect to perceptual judgments about human characters and indicate that this effect is not limited to a specific distance and does not depend on gaze.

## Author Contributions

All authors designed research; EJ and TM performed research and analyzed data; all authors wrote the paper.

## Conflict of Interest Statement

The authors declare that the research was conducted in the absence of any commercial or financial relationships that could be construed as a potential conflict of interest.

## Footnotes

1 Commonly accepted effect sizes for linear mixed models are not yet available. Thus we report partial eta square derived from data aggregated per participant and the respective condition.

## References

[B1] Alter A. L. Balcetis E. (2011). Fondness makes the distance grow shorter: desired locations seem closer because they seem more vivid. *J. Exp. Soc. Psychol.* 47 16–21.

[B2] Bailenson J. N. Blascovich J. Beall A. C. Loomis J. M. (2001). Equilibrium theory revisited?: mutual gaze and personal space in virtual environments. *Presence* 10 583–598. 10.1162/105474601753272844

[B3] Bailenson J. N. Blascovich J. Beall A. C. Loomis J. M. (2003). Interpersonal distance in immersive virtual environments. *Pers. Soc. Psychol. Bull.* 29 819–833. 10.1177/0146167203029007002 15018671

[B4] Balcetis E. (2015). Approach and avoidance as organizing structures for motivated distance perception. *Emot. Rev.* 1 1–14. 10.1177/1754073915586225

[B5] Balcetis E. Dunning D. (2009). Wishful seeing: more desired objects are seen as closer. *Psychol. Sci.* 21 147–152. 10.1177/0956797609356283 20424036

[B6] Bardi L. Di Giorgio E. Lunghi M. Troje N. F. Simion F. (2015). Walking direction triggers visuo-spatial orienting in 6-month-old infants and adults: an eye tracking study. *Cognition* 141 112–120. 10.1016/j.cognition.2015.04.014 25978184

[B7] Bhalla M. Proffitt D. R. (1999). Visual–motor recalibration in geographical slant perception. *J. Exp. Psychol. Hum. Percept. Perform.* 25 1076–1096. 10464946 10.1037//0096-1523.25.4.1076

[B8] Bosbach S. Prinz W. Kerzel D. (2004). A simon effect with stationary moving stimuli. *J. Exp. Psychol. Hum. Percept. Perform.* 30 39–55. 14769067 10.1037/0096-1523.30.1.39

[B9] Cole S. Balcetis E. Dunning D. (2013). Affective signals of threat increase perceived proximity. *Psychol. Sci.* 24 34–40. 10.1177/0956797612446953 23160204

[B10] Durgin F. H. Baird J. A. Greenburg M. Russell R. Shaughnessy K. Waymouth S. (2009). Who is being deceived? The experimental demands of wearing a backpack. *Psychon. Bull. Rev.* 16 964–969. 10.3758/PBR.16.5.964 19815806

[B11] Felipe N. J. Sommer R. (1966). Invasions of personal space. *Soc. Probl.* 14 206–214. 10.1525/sp.1966.14.2.03a00080

[B12] Firestone C. Scholl B. J. (2014). “Top-down” effects where none should be found: the El Greco fallacy in perception research. *Psychol. Sci.* 25 38–46. 10.1177/0956797613485092 24297777

[B13] Firestone C. Scholl B. J. (2015a). Cognition does not affect perception: evaluating the evidence for ‘top-down’ effects. *Behav. Brain Sci.* 10.1017/S0140525X15000965 [Epub ahead of print]. 26189677

[B14] Firestone C. Scholl B. J. (2015b). Enhanced visual awareness for morality and pajamas? Perception vs. memory in top-down effects. *Cognition* 136 409–416. 10.1016/j.cognition.2014.10.014 25547483

[B15] Hall E. T. (1968). Proxemics. *Curr. Anthropol.* 9 83–95. 10.1086/200975

[B16] Haselton M. G. Nettle D. Andrews P. W. (2005). “The evolution of cognitive bias,” in *The Handbook of Evolutionary Psychology*, ed. Buss D. M. (Hoboken, NJ: John Wiley & Sons Inc), 724–746.

[B17] Hayduk L. A. (1981). The shape of personal space: an experimental investigation. *Can. J. Behav. Sci.* 13 87–93. 10.1037/h0081114

[B18] Hecht S. (1924). The visual discrimination of intensity and the Weber-Fechner law. *J. Gen. Physiol.* 7 235–267. 10.1085/jgp.7.2.235 19872133 PMC2140693

[B19] Hommel B. Pratt J. Colzato L. Godijn R. (2001). Symbolic control of visual attention. *Psychol. Sci.* 12 360–365. 10.1111/1467-9280.00367 11554667

[B20] Iachini T. Pagliaro S. Ruggiero G. (2015). Near or far? It depends on my impression: moral information and spatial behavior in virtual interactions. *Acta Psychol.* 161 131–136. 10.1016/j.actpsy.2015.09.003 26386781

[B21] Kleck R. E. (1968). Effects of stigmatizing conditions on the use of personal space. *Psychol. Rep.* 32 111–118. 10.2466/pr0.1968.23.1.111 5685377

[B22] Kuhn G. Benson V. (2007). The influence of eye-gaze and arrow pointing distractor cues on voluntary eye movements. *Percept. Psychophys.* 69 966–971. 10.3758/BF03193934 18018978

[B23] Loomis J. M. Knapp J. M. (2003). Visual perception of egocentric distance in real and virtual environments. *Virtual Adapt. Environ.* 11 21–46.

[B24] Mohler B. J. Creem-Regehr S. H. Thompson W. B. Bülthoff H. H. (2010). The effect of viewing a self-avatar on distance judgments in an HMD-based virtual environment. *Presence* 19 230–242. 10.1162/pres.19.3.230

[B25] Patterson M. L. (1977). Interpersonal distance, affect, and equilibrium theory. *J. Soc. Psychol.* 101 205–214. 10.1080/00224545.1977.9924008

[B26] Proffitt D. R. (2006a). Embodied perception and the economy of action. *Perspect. Psychol. Sci.* 1 110–122. 10.1111/j.1745-6916.2006.00008.x 26151466

[B27] Proffitt D. R. (2006b). Distance perception. *Curr. Dir. Psychol. Sci.* 15 131–135. 10.1111/j.0963-7214.2006.00422.x

[B28] Proffitt D. R. Bhalla M. Gossweiler R. Midgett J. (1995). Perceiving geographical slant. *Psychon. Bull. Rev.* 2 409–428. 10.3758/BF03210980 24203782

[B29] Proffitt D. R. Linkenauger S. A. (2013). “Perception viewed as a phenotypic expression,” in *Action Science: Foundations of an Emerging Discipline*, eds Prinz W. Beisert M. Herwig A. (Cambridge, MA: MIT Press), 171–198.

[B30] Proffitt D. R. Stefanucci J. Banton T. Epstein W. (2003). The role of effort in perceiving distance. *Psychol. Sci.* 14 106–112. 10.1111/1467-9280.t01-1-01427 12661670

[B31] Riener C. R. Stefanucci J. K. Proffitt D. R. Clore G. (2011). An effect of mood on the perception of geographical slant. *Cogn. Emot.* 25 174–182. 10.1080/02699931003738026 21432665 PMC3298357

[B32] Schnall S. Harber K. D. Stefanucci J. K. Proffitt D. R. (2008). Social support and the perception of geographical slant. *J. Exp. Soc. Psychol.* 44 1246–1255. 10.1016/j.jesp.2008.04.011 22389520 PMC3291107

[B33] Schultz J. Bülthoff H. H. (2013). Parametric animacy percept evoked by a single moving dot mimicking natural stimuli. *J. Vis.* 13 1–19. 10.1167/13.4.15. 23525131

[B34] Shi J. Weng X. He S. Jiang Y. (2010). Biological motion cues trigger reflexive attentional orienting. *Cognition* 117 348–354. 10.1016/j.cognition.2010.09.001 20883983 PMC2967601

[B35] Stefanucci J. K. Proffitt D. R. Clore G. L. Parekh N. (2008). Skating down a steeper slope: fear influences the perception of geographical slant. *Perception* 37 321–323. 10.1068/p5796 18414594 PMC2293293

[B36] Takahashi K. Meilinger T. Watanabe K. Bülthoff H. H. (2013). Psychological influences on distance estimation in a virtual reality environment. *Front. Hum. Neurosci.* 7:580 10.3389/fnhum.2013.00580 PMC3776303 24065905

[B37] Tomasello M. (1995). “Joint attention as social cognition,” in *Joint Attention: Its Origins and Role in Development*, eds Moore C. Dunham P. (Hillsdale, NJ: Lawrence Erlbaum), 103–130.

[B38] Willis F. N. Jr. (1966). Initial speaking distance as a function of the speakers’ relationship. *Psychon. Sci.* 5 221–222. 10.3758/BF03328362

